# The impact of true knot of umbilical cord on obstetric outcomes—true or not?

**DOI:** 10.1007/s00404-025-08110-7

**Published:** 2025-07-11

**Authors:** Shay Porat, Doron Kabiri, Gilad Karavani, Hagai Amsalem, Michal Lipschuetz, Joshua I. Rosenbloom

**Affiliations:** 1 Department of Obstetrics and Gynecology, Hadassah Ein Kerem Medical Center, Faculty of Medicine, Hebrew University, Jerusalem, Israel; 2 Department of Obstetrics and Gynecology, Hadassah Mt Scopus Medical Center, Faculty of Medicine, Hebrew University, Jerusalem, Israel; 3 Faculty of Medicine, Henrietta Szold Hadassah Hebrew University School of Nursing, Jerusalem, Israel

**Keywords:** True knot, Umbilical cord, Perinatal mortality, Perinatal death, Nuchal cord, SGA

## Abstract

**Objectives:**

To quantify the risk of true cord of the umbilical cord for perinatal death and identify additional risk factors.

**Methods:**

This retrospective study included singleton deliveries between 24 and 42 weeks of gestation that took place between 2003 and 2017 in two medical centers. For patients with true knot, data regarding the number of cord knots as well as the location and number of loops of cord were obtained. The rest of the birth data set comprised the control group. The primary outcome was fetal demise. Secondary outcomes included mode of delivery and perinatal outcomes.

**Results:**

The final data set included 139,458 deliveries, of which 364 cases (0.26%) comprised the study group (true knot group) and 139,094 cases (99.74%) the control group. Higher rated of unfavorable outcomes were found among cases than controls, including perinatal death, delivery mode, lower Apgar and cord blood umbilical artery pH as well as higher rates of NICU admission and perinatal death. Multivariate analysis showed that true knot of cord (aOR 15.46, 95% CI 9.30–25.70) was a strong predictor of perinatal death. Analysis of predictors of perinatal death within the study group showed that only four or more nuchal loops of cord was an independent predictor (four loops OR 13.40 95% CI 1.12–160.34).

**Conclusions:**

True knot of the umbilical cord is a strong predictor of perinatal death. Fetuses with true knot of cord and four or more nuchal cord loops are at significantly increased risk of perinatal death. If diagnosed before onset of labor, delivery before 37 weeks may prevent perinatal death.

**Supplementary Information:**

The online version contains supplementary material available at 10.1007/s00404-025-08110-7.

## Introduction

True knot of the umbilical cord is found in 0.3–2.1% of pregnancies. Among the known risk factors are longer cord, male sex, older maternal age, multiparity, previous miscarriages, prolonged pregnancy, polyhydramnios maternal diabetes, pre-existing hypertension and obesity [2–5]. Some studies have associated true knot in the umbilical cord with complications before or during labor: intra-uterine fetal demise (IUFD) [1–8], meconium staining of amniotic fluid [3, 6, 8], increased rate of cesarean delivery (CD) [3, 6], higher rate of small for gestational age (SGA) neonates [7, 8], lower Apgar scores at 1 min [2, 6, 7] and 5 min [5–7] and increased rate of neonatal intensive care unit (NICU) admission [5, 7]. However, only two studies addressed the gestational age at risk for IUFD [4, 5]. Moreover, except for increased rate of fetal demise on which all studies agree, there is some disagreement or even discrepancy regarding other complications. Finally, although Linde et al. investigated the relationship between true knot and cord entanglement, no study has systematically analyzed the combined effect of true cord knot and number of umbilical cord loops around the neck, body or limbs [4]. Therefore, in this study we aimed to evaluate the impact of the true knot of the umbilical cord on perinatal fetal death and identify additional risk factors for this outcome. Secondarily we aimed to assess its impact on additional obstetric adverse outcomes in our population.

## Materials and methods

### Patient population

This retrospective study included all singleton pregnancies between 24 and 42 weeks of gestation that took place between 2003 and 2017 in either one of the two Hadassah hospital campuses in Jerusalem, Israel. Exclusion criteria included: termination of pregnancy, incomplete or inconsistent records, fetuses with major chromosomal or structural malformations (complete list in appendix A), deliveries outside of the hospital (home, vehicle or ambulance), and clinically significant placental abruption.

A textual search strategy was applied on midwife birth summary, physician birth summary and cesarean section note. The search included all records with one or more of the following words in Hebrew and/or English “knot”, “knots”, “true”. Then each birth summary from the set that contained the above terms was individually read and assessed. Data regarding the number of cord knots and location and number of loops of cord was manually obtained. This set of records comprised the study group (true knot group). The rest of the entire birth data set comprised the control group.

The retrieved data set included maternal demographic data, data relating to labor onset and course, and neonatal outcome measures.

The primary outcome was perinatal death rate. We chose perinatal death rather the fetal death, because we presumed that after excluding all major malformations, most if not all of the intrapartum and immediate neonatal death (during the first week of life) were a direct result of delivery complications. Secondary outcomes were mode of delivery (vaginal, instrumental, cesarean), cesarean urgency (elective, urgent, emergent), non-reassuring fetal monitor as an indication for urgent CD in pre-labor patients and in patients undergoing trial of labor, meconium staining grading of amniotic fluid (slight, mild, prominent, thick), means of Apgar scores in 1 and 5 min, means of birthweight (grams), percentage rates of small for gestational age (below 10th, 5th and 1st percentile according to Israeli birthweight reference curves [9]), rates of cord umbilical artery blood pH ≤ 7.1 or ≤7.0 or ≤6.9, and NICU admission rates.

To analyze the perinatal death rate as a function of gestational week we calculated the perinatal death rate per 10,000 ongoing pregnancies for each specific gestational week. The denominator included all women with a fetus still in utero at the gestational week being studied, and the numerator included the number of fetal deaths during the same gestational week.

### Statistical analysis

Patient characteristics and results are described as proportions for categorical variables and means and standard deviations for continuous variables. Comparisons between groups were assessed using the chi-square test or Fisher’s exact test (when appropriate) for categorical variables. Continuous variables were compared by *t* test with or without the assumption of equal variance according to Levene’s test for equality of variances.

A univariate binary logistic regression analysis was performed and risk factors for the primary outcome  were identified. Then a multivariate binary logistic regression model was built based on the univariate analysis to identify independent risk factors. Then, we performed a second univariate analysis analyzing predictors for perinatal death only in the study group (patients with true knot). Unfortunately, we did not have enough statistically significant predictors to perform multi-variable binary logistic regression within this subgroup. The data were analyzed using Software Package for Statistics and Simulation (IBM SPSS version 29, IBM Corp, Armonk, NY).

A *P* value <0.05 was considered statistically significant.

### Ethics

This study was approved by the hospital’s Helsinki committee. Approval # HMO-0436-21. A waiver of informed consent was obtained due to the retrospective nature of the study.

## Results

**Fig. 1:**
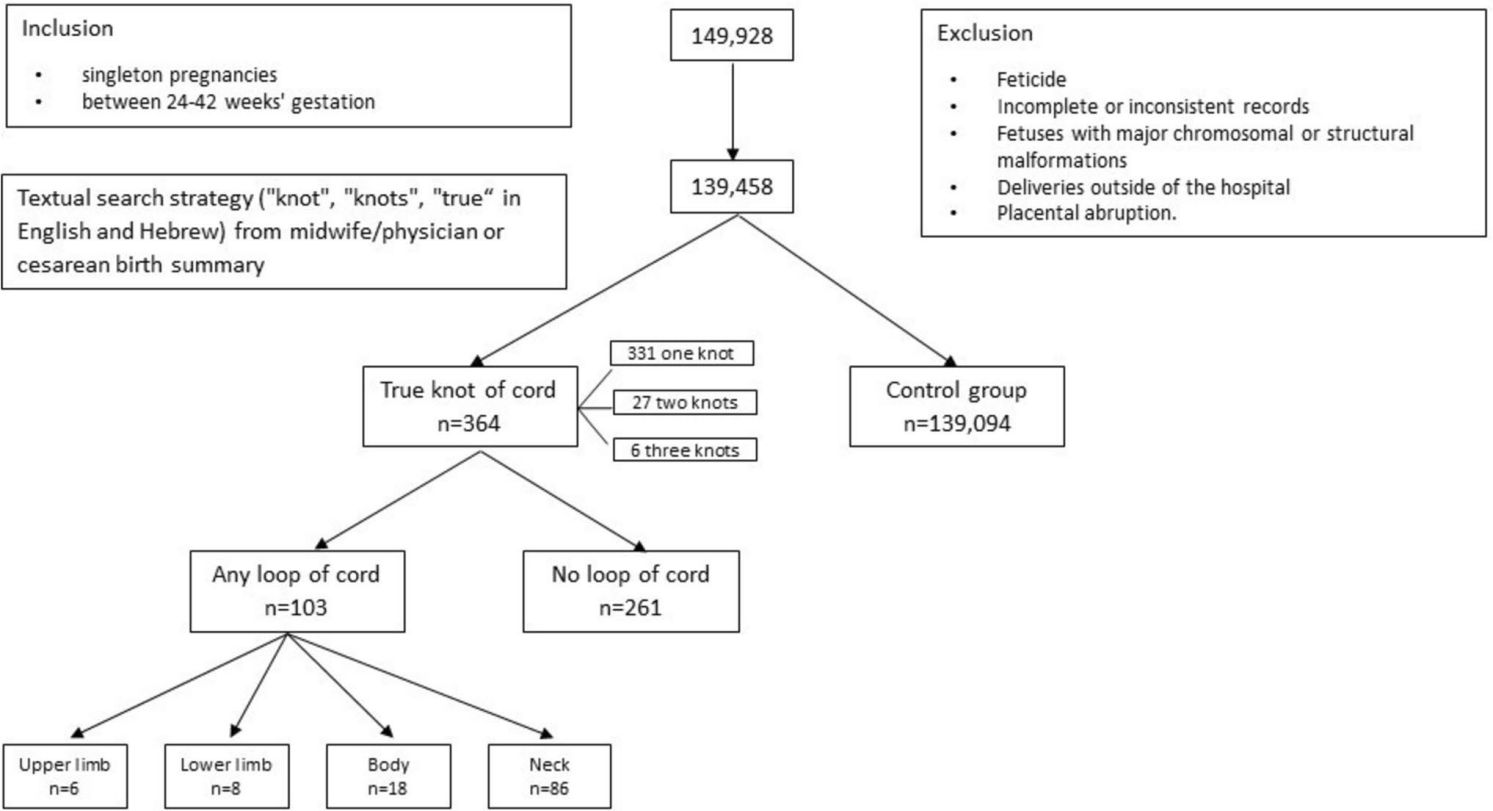
Flowchart describing study design

The Hadassah Medical Organization electronic births database included 142,459 records that fulfilled inclusion criteria between the years 2003 and 2017. After application of exclusion criteria, 139,458 records were eligible for the study. The study group included 364 cases (0.26%) with umbilical true knot of cord and the control group included 139,094 cases (99.74%) without true knot. Of the study group 331 cases (90.9%) had one true knot, 27 (7.4%) had two knots and 6 cases (1.6%) had three knots. The study group was further segregated into cases with or without one or more cord loops. Altogether there were 103 cases (28.3%) with one or more cord loops: 86 cases (83.5%) with cord around the neck, 18 (17.5%) around the body, 6 cases (5.8%) around upper limb and 8 cases (7.8%) around the lower limb. The total exceeds 100%, as there were several patients with cord loops around more than one body part. There were 261 cases (71.7%) without any cord loop. Study design is shown in Fig. 1.

In terms of baseline characteristics both groups differed significantly in almost all tested parameters except for the proportion of patients with hypertensive disorders. (Results are shown in Table 1). Patients in the study group tended to be older, deliver at a slightly earlier gestational age, have slightly higher gravidity and parity, and to have a lower proportion of primiparity. They had higher rate of gestational diabetes, were more likely to smoke and less likely to test positive for group B streptococcus. Finally, they had a significantly higher proportion of male fetuses.

**Table 1:** Baseline characteristics

Parameter	True knot of cord		Control		*P* value
	*N*	Mean (SD) or proportion (%)	*N*	Mean (SD) or proportion (%)	
Maternal age	363	31.87 (5.83)	138,415	29.92 (5.61)	<0.001
Gestational age	364	38.67 (2.06)	139,094	39.08 (1.72)	<0.001
Gravidity	364	3.89 (2.65)	139,079	3.39 (2.43)	<0.001
Parity	364	2.27 (2.11)	139,078	1.88 (1.99)	0.001
Primiparity	72	19.80%	38,526	27.70%	0.001
Diabetes					
No diabetes	348	95.60%	136,038	97.80%	0.033
Type 1	0	0.00%	44	0.03%	
Type 2	0	0.00%	12	0.01%	
Gestational	16	4.40%	3000	2.20%	
Hypertension					
No hypertension	362	99.50%	138,777	99.80%	0.422
Chronic	0	0.00%	9	0.01%	
Gestational	2	0.50%	255	0.20%	
Preeclampsia	0	0.00%	53	0.04%	
Smoking					
Yes	21	5.80%	4673	3.40%	<0.001
No	327	89.80%	108,837	78.20%	
Missing data	16	4.40%	25,584	18.40%	
GBS					
Positive	26	7.10%	13,867	10.00%	0.001
Negative	116	31.90%	33,113	23.80%	
Not tested	222	61.00%	92,114	66.20%	
Male fetus	221	60.70%	71,400	51.30%	<0.001

*GBS* group B Streptococcus

The primary outcome of perinatal death was significantly higher in the study group (4.7% vs. 0.2%, *p* < 0.001).

Delivery characteristics were also significantly different between the study and control groups, as shown in Table 2. All studied outcomes differed significantly between groups. Patients in the study group were more likely to have either induction of labor or CD with no TOL rather than spontaneous onset of labor. CD as the mode of delivery was strikingly higher in the study group (42.3% vs. 17.1%, *p* < 0.001), the difference remained also when analyzing only the subgroup of patients than underwent a TOL (20.5% in the study group vs. 5.5% in the control group, *p* < 0.001). The CDs in the study group were generally more urgent than in the control group (72.7% urgent/emergent CD in the study group vs. 55.7% in the control group, *p* < 0.001), and the indication of NRFHR was significantly more frequent (29% in the study group vs. 11% in the control group in non-TOL subgroup, *p* < 0.001 and 59.3% in the study group vs. 42.5% in the control group in TOL subgroup, *p* = 0.013). Another aspect of fetal reaction to the stress of labor is the frequency and amount of meconium staining of amniotic fluid. In this regard the study group had almost double the rate of prominent staining (11.9% vs. 5.1%, *p* < 0.001) and more than triple the rate of heavy staining (5.0% vs. 1.5%, *p* < 0.001).

**Table 2:** Delivery outcomes

Parameter	True knot of cord		Control		*P* value
	*N*	Mean (SD) or proportion (%)	*N*	Mean (SD) or proportion (%)	
Labor onset^~^					
Spontaneous	196	53.8%	100,999	74.3%	<0.001
Induction	68	18.7%	20,525	15.1%	
CD no TOL	100	27.5%	14,452	10.6%	
General anesthesia	25	6.9%	3,539	2.5%	<0.001
Regional anesthesia	243	66.8%	83,367	59.9%	0.008
Mode of delivery (all patients)					
Spontaneous	183	50.3%	106,254	76.4%	<0.001
Instrumental	27	7.4%	9,063	6.5%	
CD (planned + unplanned)	154	42.3%	23,777	17.1%	
Mode of delivery (only TOL)^~^					
Spontaneous	183	69.3%	105,877	87.1%	<0.001
Instrumental	27	10.2%	9,020	7.4%	
CD	54	20.5%	6,627	5.5%	
CD urgency^*^					
Elective	42	27.3%	10,278	44.3%	<0.001
Urgent	103	66.9%	12,308	53.1%	
Emergent	9	5.8%	604	2.6%	
NRFHR as an indication					
CD no TOL	29	29%	1,590	11.0%	<0.001
CD after TOL	32	59.3%	2,817	42.5%	0.013
Meconium staining					
None	190	73.1%	98,340	84.8%	<0.001
Slight	26	10.0%	10,065	8.7%	
Prominent	31	11.9%	5,861	5.1%	
Thick	13	5.0%	1,716	1.5%	

*CD* Cesarean delivery, *TOL* trial of labor, *NRFHR* non-reassuring fetal heart rate

~ 3482 records with missing data

^*^ 587 records with missing data

Neonatal outcomes were also highly different between groups, as shown in Table 3. The study group’s average 1-min and 5-min Apgar scores were lower (8.24 vs. 8.84 at 1 min, *p* < 0.001 and 9.27 vs. 9.85 at 5 min, *p* < 0.001), and a larger proportion had cord blood umbilical artery pH less than 7.0 (1.5% vs. 0.2%, *p* = 0.035). Interestingly, the study group’s average birthweight was lower than the control group, and this difference was also reflected in the proportion of neonates that were SGA less than the 10th percentile (11.3% vs. 7.1%, *p* = 0.002) and less than the 5th percentile (4.9% vs. 3.1%, *p* = 0.038) according to Israeli standards. Almost twice as many neonates in the study group were admitted to the NICU (4.7% vs. 2.4%, *p* = 0.005).

**Table 3:** Neonatal outcomes

Parameter	True knot of cord		Control		*P* value
	*N*	Mean (SD) or proportion (%)	*N*	Mean (SD) or proportion (%)	
Apgar at 1 min	364	8.24 (2.13)	139,090	8.84 (0.80)	<0.001
Apgar at 5 min	364	9.27 (2.17)	139,094	9.85 (0.68)	<0.001
pH					
pH ≤ 7.1	3	2.2%	457	1.3%	0.261
pH ≤ 7.0	2	1.5%	74	0.2%	0.035
pH ≤ 6.9	0	0%	12	0.03%	1
Birthweight	364	3,166 (578)	139,094	3,252 (492)	0.005
SGA < 10th percentile	41	11.3%	9813	7.1%	0.002
SGA < 5th percentile	18	4.9%	4265	3.1%	0.038
SGA < 1st percentile	1	0.3%	564	0.4%	1
Need for ICU	17	4.7%	2886	2.4%	0.005
Perinatal death	17	4.7%	347	0.2%	<0.001

*SGA* small for gestational age, was defined according to Israeli standard

While evaluating the predictors of perinatal death, in a univariate analysis of the entire cohort demonstrated that true knot of cord, older maternal age, previous CD, earlier birth year, Arab ethnicity, being SGA less than the 10th, 5th and 1st percentile are all positive predictors of perinatal death (Table 4). Multivariate analysis showed that true knot of cord (aOR 15.46, 95% CI 9.30–25.70), older maternal age (aOR 1.03, 95% CI 1.01–1.05), Arab ethnicity (aOR 1.61, 95% CI 1.22–2.14) compared to Jewish ethnicity, being SGA less than the 1st (aOR 16.48, 95% CI 10.32–26.33), 1st–5th percentile (aOR 4.17, 95% CI 2.90–5.98) and 5th–10th percentile (aOR 2.73, 95% CI 1.89–3.93) were found as independent predictors of perinatal death.

**Table 4:** Predictors of perinatal death from the uni- and multi-variable binary logistic regression analysis

Parameter	Univariate analysis		Multivariate analysis	
	OR(95% CI)	*P* value	aOR(95% CI)	*P* value
True knot	19.59 (11.90–32.24)	<0.001	15.46 (9.30–25.70)	<0.001
Maternal age	1.03 (1.01–1.04)	0.003	1.03 (1.01–1.05)	<0.001
Number of previous Cesarean sections	1.20 (1.04–1.37)	0.011		NS
Delivery year	0.95 (0.92–0.97)	<0.001		NS
Ethnicity				
Jewish (reference)	1		1	
Arab	1.58 (1.20–2.07)	0.001	1.61 (1.22–2.14)	<0.001
Other	1.07 (0.47–2.40)	0.877	1.04 (0.46–2.37)	0.926
Gestational age at delivery	0.65 (0.64–0.67)	<0.001	0.65 (0.63–0.66)	<0.001
Birthweight percentile				
<1	16.63 (10.47–26.42)	<0.001	16.48 (10.32–26.33)	<0.001
1–5	4.31 (3.03–6.15)	<0.001	4.17 (2.9–5.98)	<0.001
5–10	2.71 (1.88–3.89)	<0.001	2.73 (1.89–3.93)	<0.001
10–90	1	–	–	–
90–95				
95–99				
>99				
SGA (1st–5th percentile)		<0.001	4.17 (2.90–5.98)	<0.001
SGA (5th–10th percentile)	2.71 (1.88–3.89)	<0.001	2.73 (1.89–3.93)	<0.001

Birthweight 10–90th percentile was the reference for all other birthweight categories. *OR* odds ratio, *aOR* adjusted odds ratio, *SGA* small for gestational age, *CI* confidence interval, *NS* non-significant

Interestingly, only four or more nuchal loops of cord were found to be an independent predictor for perinatal death within the true knot group (four loops OR 13.4 95% CI 1.12–160.34, five loops OR 26.80, 95% CI 1.56–459.98, as compared to no loops).

**Fig. 2:**
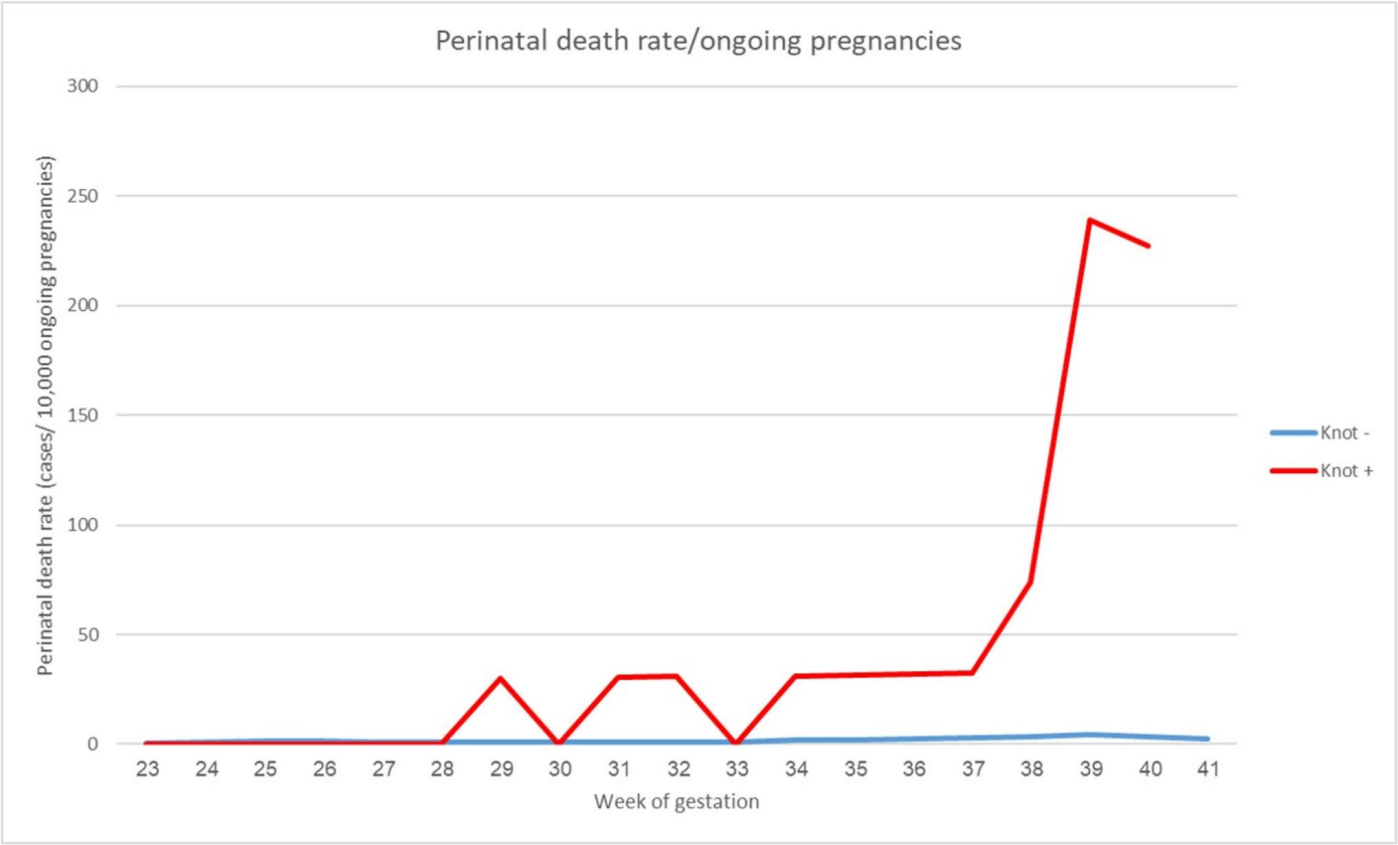
Perinatal death rate against gestational week, expressed as cases per 10,000 ongoing pregnancies

Analysis of the timing of perinatal death shows that there was an increased likelihood of perinatal death after 37 weeks of gestation in cases vs. controls, as shown in Fig. 2.

## Discussion

In this study of over 100,000 births at two tertiary hospitals, we found that a true knot of the umbilical cord was significantly associated with multiple adverse obstetric outcomes, including perinatal death, CD (particularly for non-reassuring fetal heart rate tracing), meconium-stained amniotic fluid, low APGAR scores, acidemia, SGA, and NICU admission. These risks were augmented in the presence of multiple cord loops. Both the absolute risk of perinatal death (4.7%) and as well as the 15-times higher odds of perinatal death in the study group relative to control group warrant close attention.

The incidence of true knot in this study, *n* = 364 (0.26%), was slightly lower than the range reported in the literature, with most recent cohorts reporting a risk of approximately 1% [1–8]. Our finding that a true knot of the umbilical cord is associated with IUFD is similar to findings from previous studies [1–8], although in this study, the magnitude of the risk was somewhat higher. This may be due to differences in our eligibility criteria, underlying demographic differences in our study population, or under-reporting. Although it has been previously reported that the combination of true knot and a nuchal cord is associated with increased risk [10], the additional risk conferred by multiple (4 or more) cord loops is a novel finding that requires confirmation in further studies. A recent meta-analysis on abnormal umbilical cords demonstrated an increased risk of IUFD in the presence of a true knot, although the possible additive effect of cord loops was not discussed [1].

Several hypotheses have emerged to explain the marked increase in intrauterine fetal death rate at the 37th and 38th weeks of gestation in women with a true knot of the umbilical cord. One theory considers late-term placental insufficiency, where the growing fetal demand for nutrients might exceed the placental supply, potentially leading to fetal demise. Another hypothesis suggests that umbilical cord compression could contribute significantly to the observed fetal demise. As the fetus grows and the uterine space becomes constrained, the true knot may be subjected to increased compression, leading to decreased blood flow and subsequent risk of IUFD. This situation may be further aggravated by hemodynamic changes near term, with elevated blood pressure within the cord due to augmented blood flow potentially intensifying compression forces on cord vessels. The increased incidence of IUFD at term might also be associated with the intensification of uterine contractions, which could exert more pressure on the umbilical cord knot. Combined with shifts in the fetal cardiac output that less favor the placenta despite rising fetal demand, these factors may contribute to the observed outcomes [4, 5]. Finally, surveillance bias provides another potential explanation. The higher incidence of IUFD detected during these specific weeks may be due, in part, to women presenting for routine check-ups or delivery, leading to increased detection of IUFD. Hence, the observed surge in IUFD cases may reflect increased detection rather than a genuine increase in incidence. Women may be more likely to present for routine check-ups or delivery in this gestational period, which could lead to increased detection of IUFD. The seeming surge in IUFD cases may, therefore, in part, reflect increased detection rather than an actual increase in incidence. These various hypotheses may pose explanations for this complex phenomenon and call for further research for validation.

Besides for perinatal mortality, the finding that true knot is associated with other adverse outcomes has been found in most, but not all, studies [2–4, 6, 7]. Interestingly, although we found a higher rate of cesarean delivery for non-reassuring fetal heart tracing, the one study that specifically studied fetal heart tracing characteristics in the presence of a true knot did not find any increase in repetitive late decelerations in these patients [11]. We were unable to evaluate the characteristics of the specific tracings that led to cesarean delivery in our study group; however, this question bears further study.

This study suggests that, given that true knot is a stronger risk factor for perinatal death than that for known risk factors, such as previous stillbirth [12], type 1 or 2 diabetes [13], or fetal growth restriction [14], when a true knot is found intensive antenatal surveillance may be warranted. Ultrasound diagnosis of true knot is feasible and has been demonstrated in several case reports and cohort studies [15–20]. Although diagnosable on 2D ultrasound, 3D with power Doppler may aid in the diagnosis. Classically, it presents with the “hanging noose sign” [16, 17]; in addition, the tightness of the knot can be assessed using 4D ultrasound [17] or by noting the absence of amniotic fluid within the loop of the knot [15]. However, there are some pitfalls in diagnosis, as multiple loops close together may resemble a knot [21].

Although the feasibility of ultrasound screening for true knot has been demonstrated [15], it is not known whether it is cost-effective to screen all patients for true knot (and if so, when and at what intervals), and what the optimal management of these patients is. Specifically, it is not known whether antenatal fetal monitoring or ultrasound can reduce the risk of stillbirth and if induction of labor at a certain point reduces the risk of perinatal mortality. Large-scale prospective studies addressing diagnosis and management of true knot are needed. Until such studies are conducted, we suggest that an incidentally noted ultrasound diagnosis of true knot be managed according to a protocol similar to that of Weissmann-Brenner et al. [15], including serial ultrasound monitoring and induction of labor at 37–38. On the other hand, earlier delivery (prior to 37 weeks) may further reduce the risk of perinatal death, since in this study, we found that the risk of IUFD increased from 37 weeks in patients with a true knot.

Strengths of this study include the large number of patients with a true knot, the meticulous extraction of relevant clinical and demographic data, and the information on cord loops in addition to a true knot. The large sample size and varied patient demographic characteristics increase the generalizability of our findings to other settings. In addition, we were able to analyze the risk of perinatal death as a function of gestational week.

At the same time, there are some limitations to consider. First, we do not know if some of the patients with true knot or cord loops had antenatal diagnosis and if so, how this may have impacted obstetric decision-making.

Second, the study was retrospective and relied on free-text documentation of true knot and cord loops in the birth summary. Therefore, there is a possibility of misclassification bias. In particular, there may have been under-documentation of true knots in otherwise uneventful births, since after an adverse birth outcome the search and documentation of potential causes, including a true knot, may lead to more careful documentation of this finding. If indeed the true prevalence of true knot in our cohort was closer to 1% (in accordance with the literature and 4 times higher than the rate in this study), then the magnitude of our findings would be reduced accordingly. Specifically, if we assume that there were four times as many patients with true knot, the rate of perinatal mortality in patients with true knot in this study would have been 1.2% compared to 0.25% in patients without true knot, still a significantly increased risk.

## Conclusions

A 1952 case report on true knot causing IUFD concluded “Unfortunately, establishing the diagnosis is often of merely academic interest, for intervention can rarely be timely enough to produce a living infant” [22]. However, with the advent of ultrasound diagnosis and fetal monitoring, this caveat is no longer true. True knot of cord is a strong independent predictor of perinatal death. The addition of multiple cord loops to a true knot further increases this risk. Antenatal diagnosis and meticulous follow-up could potentially reduce these risks, although further prospective studies are needed.

## Supplementary Information

Below is the link to the electronic supplementary material.

Supplementary file 1 (DOCX 12 KB)

## Data Availability

Data will not be made available.

## Abbreviations

NICU: Neonatal intensive care unit
SGA: Small for gestational age
OR: Odds ratio
aOR: Adjusted odds ratio
IUFD: Intrauterine fetal demise
NRFHR: Non-reassuring fetal heart rate
CD: Cesarean delivery
TOL: Trial of labor

## References

[1] Hayes DJL, Warland J, Parast MM, Bendon RW, Hasegawa J, Banks J et al (2020) Umbilical cord characteristics and their association with adverse pregnancy outcomes: a systematic review and meta-analysis. PLoS ONE 15(9):e0239630 32970750 10.1371/journal.pone.0239630 PMC7514048

[2] Airas U, Heinonen S (2002) Clinical significance of true umbilical knots: a population-based analysis. Am J Perinatol 19(3):127–132 12012287 10.1055/s-2002-25311

[3] Hershkovitz R, Silberstein T, Sheiner E, Shoham-Vardi I, Holcberg G, Katz M et al (2001) Risk factors associated with true knots of the umbilical cord. Eur J Obstet Gynecol Reprod Biol 98(1):36–39 11516797 10.1016/s0301-2115(01)00312-8

[4] Weissmann-Brenner A, Meyer R, Domniz N, Levin G, Hendin N, Yoeli-Ullman R et al (2022) The perils of true knot of the umbilical cord: antepartum, intrapartum and postpartum complications and clinical implications. Arch Gynecol Obstet 305(3):573–579 34405285 10.1007/s00404-021-06168-7

[5] Linde LE, Rasmussen S, Kessler J, Ebbing C (2018) Extreme umbilical cord lengths, cord knot and entanglement: risk factors and risk of adverse outcomes, a population-based study. PLoS ONE 13(3):e0194814 29584790 10.1371/journal.pone.0194814 PMC5870981

[6] Lichtman Y, Wainstock T, Walfisch A, Sheiner E (2020) The significance of true knot of the umbilical cord in long-term offspring neurological health. J Clin Med 10(1):123 33396487 10.3390/jcm10010123 PMC7796317

[7] Raisanen S, Georgiadis L, Harju M, Keski-Nisula L, Heinonen S (2013) True umbilical cord knot and obstetric outcome. Int J Gynaecol Obstet 122(1):18–21 23523334 10.1016/j.ijgo.2013.02.012

[8] Bas Lando M, Sela HY, Helman S, Shapira E, Grisaru-Granovsky S, Rottenstreich M (2025) Adverse perinatal outcomes associated with true knot of the umbilical cord: a multicenter retrospective study. Am J Perinatol 10.1055/a-2553-9200 40064311

[9] Dollberg S, Haklai Z, Mimouni FB, Gorfein I, Gordon ES (2005) Birth weight standards in the live-born population in Israel. Israel Med Assoc J 7(5):311–314 15909464

[10] Sherer DM, Dalloul M, Ward K, Nakagawa J, Joseph I, Grube S et al (2017) Coexisting true umbilical cord knot and nuchal cord: possible cumulative increased risk of adverse perinatal outcome. Ultras Obstet Gynecol 50(3):404–405 10.1002/uog.17389 27997052

[11] Carter EB, Chu CS, Thompson Z, Tuuli MG, Macones GA, Cahill AG (2018) True knot at the time of delivery: electronic fetal monitoring characteristics and neonatal outcomes. J Perinatol 38(12):1620–1624 30323323 10.1038/s41372-018-0250-4 PMC6279587

[12] Lamont K, Scott NW, Gissler M, Gatt M, Bhattacharya S (2022) Risk of recurrent stillbirth in subsequent pregnancies. Obstet Gynecol 139(1):31–40 34856561 10.1097/AOG.0000000000004626

[13] Mackin ST, Nelson SM, Wild SH, Colhoun HM, Wood R, Lindsay RS et al (2019) Factors associated with stillbirth in women with diabetes. Diabetologia 62(10):1938–1947 31353418 10.1007/s00125-019-4943-9 PMC6731193

[14] Unterscheider J, Daly S, Geary MP, Kennelly MM, McAuliffe FM, O’Donoghue K et al (2013) Optimizing the definition of intrauterine growth restriction: the multicenter prospective PORTO Study. Am J Obstet Gynecol 208(4):290 e1–6 10.1016/j.ajog.2013.02.007 23531326

[15] Weissmann-Brenner A, Domniz N, Weissbach T, Mazaki-Tovi S, Achiron R, Weisz B et al (2022) Antenatal detection of true knot in the umbilical cord—how accurate can we be? Ultraschall Med 43(3):298–303 32674187 10.1055/a-1205-0411

[16] Ramon YCCL, Martinez RO (2004) Prenatal diagnosis of true knot of the umbilical cord. Ultras Obstet Gynecol 23(1):99–100 10.1002/uog.900 14971012

[17] Ramon y Cajal CL, Martinez RO (2006) Four-dimensional ultrasonography of a true knot of the umbilical cord. Am J Obstet Gynecol 195(4):896–898 17000232 10.1016/j.ajog.2006.05.044

[18] Singh C, Kotoch K (2020) Prenatal diagnosis of true knot of the umbilical cord. J Obstet Gynaecol Canada 42(9):1065–1066 10.1016/j.jogc.2019.03.003 31402268

[19] Gembruch U, Baschat AA (1996) True knot of the umbilical cord: transient constrictive effect to umbilical venous blood flow demonstrated by Doppler sonography. Ultras Obstet Gynecol 8(1):53–56 10.1046/j.1469-0705.1996.08010053.x 8843621

[20] Clerici G, Koutras I, Luzietti R, Di Renzo GC (2007) Multiple true umbilical knots: a silent risk for intrauterine growth restriction with anomalous hemodynamic pattern. Fetal Diagn Ther 22(6):440–443 17652933 10.1159/000106351

[21] Hasbun J, Alcalde JL, Sepulveda W (2007) Three-dimensional power Doppler sonography in the prenatal diagnosis of a true knot of the umbilical cord: value and limitations. J Ultras Med 26(9):1215–1220 10.7863/jum.2007.26.9.1215 17715316

[22] Peterson WF (1952) True knot of umbilical cord resulting in fetal death; report of a case. J Am Med Assoc 150(10):1009–1010 12990340 10.1001/jama.1952.63680100008014d

