# Supplementary material for: The impact of true knot of umbilical cord on obstetric outcomes—true or not?
Source: Arch Gynecol Obstet. 2025 Jul 11;312(4):1175–83. doi: 10.1007/s00404-025-08110-7 (PMC12414008; doi:10.1007/s00404-025-08110-7)
Supplement: Supplementary file 1 — (DOCX 12 KB) [file 404_2025_8110_MOESM1_ESM.docx]

Appendix A

List of excluded diagnoses :

CNS :

Hydrocephalus

Agenesis of corpus callosum (partial or complete)

Dandy-Walker malformation

Myelocele

Encephalocele

Spina bifida

Heart:

Hypoplastic left/right heart

Tetralogy of Fallot

Double outlet right ventricle

Transposition of great arteries

Right aortic arch

Atrio-ventricular septal defect

Aortic coarctation

Tricuspid atresia

Truncus arteriosus

Lungs:

Sequestration

Congenital pulmonary airway malformation

Gastrointestinal tract:

Double bubble

Suspected esophageal atresia

Bowel obstruction

Gastroschisis

Omphalocele

Facial:

Cleft lip and/or palate

Micrognathia

Urinary tract:

Severe hydronephrosis

Multicystic kidneys

Other:

Hydrops fetalis

Severe polyhydramnios

Clubfoot

Hemivertebra

Scoliosis

Polydactyly

Congenital diaphragmatic hernia

Dysmorphic/Dysmorphism

Genetic:

Trisomy

Aneuploidy

Syndrome
